# Reduced atherogenic indices in prepubertal girls with precocious adrenarche born appropriate for gestational age in relation to the conundrum of DHEAS

**DOI:** 10.1530/EC-12-0059

**Published:** 2012-11-19

**Authors:** Ahmet Uçar, Nurçin Saka, Firdevs Baş, Nihal Hatipoğlu, Rüveyde Bundak, Feyza Darendeliler

**Affiliations:** Pediatric Endocrine Unit, Istanbul Medical Faculty Istanbul University 34093, Fatih, Istanbul Turkey; 1 Pediatric Endocrine Unit, Erciyes Medical Faculty Erciyes University KayseriTurkey

**Keywords:** precocious adrenarche, DHEAS, insulin sensitivity, body mass index, waist circumference, atherogenic index

## Abstract

**Context:**

An association between low birth weight, insulin resistance (IR), dyslipidemia, and atherogenesis has been shown in girls with precocious adrenarche (PA).

**Objective:**

To evaluate whether girls with PA born appropriate for gestational age (AGA) have increased risk for metabolic complications at initial evaluation.

**Design/methods:**

We conducted a cross-sectional study on 69 AGA born girls with PA (mean (±s.d.) age 7.1±1 years) and 45 body mass index (BMI)- and waist circumference (WC)-matched prepubertal peers born AGA (mean (±s.d.) 7.5±1.9 years). A standard 2-h oral glucose tolerance test with insulin sampling was performed. Fasting plasma lipids and high-sensitivity C-reactive protein were analyzed, and blood pressure was recorded. Insulin sensitivity (IS) index (ISI_comp_), homeostasis model assessment of IR, and atherogenic index (AI) (triglycerides/high-density lipoprotein cholesterol) were calculated.

**Setting:**

The study was performed at University Hospital.

**Results:**

AI was significantly lower in girls with PA than in controls (*P*<0.001), and it was correlated with BMI SDS (*r*=0.44, *P*=0.001) and WC (*r*=0.39, *P*=0.001). The significant correlation of AI with ISI_comp_ (*r*=−0.38, *P*=0.001) disappeared after correcting for BMI (*r*=−0.185, *P*=0.16). Multivariate regression analysis revealed that DHEAS was the only significant parameter influencing AI in girls with born AGA (*R* ^2^=0.475 *β*=−0.018, *P*=0.0001).

**Conclusions:**

Metabolic screening in prepubertal AGA born girls with PA may yield favorable lipid profiles. AI in girls with PA is increased in relation to decreasing IS and increasing BMI and WC. DHEAS seems to have the most significant effect on AI.

## Introduction

Precocious adrenarche (PA) refers to the concurrent presence of adrenal androgen levels increased above the age- and sex-specific range and clinical signs of an increased androgen action before the age of 8 years in girls (1). It is biochemically characterized by serum DHEAS concentrations over 40 μg/dl. The etiology of PA is not clear. Relative deficiency of 3β-hydroxysteroid dehydrogenase, early activation of adrenarche by extra- and intra-adrenal factors that are yet uncertain, and genetic factors have been proposed (2, 3). PA has been associated with type 2 diabetes, coronary arterial disease (CAD), hypertension, and metabolic syndrome in adulthood (4, 5). These long-term complications have been linked to low birth weight (BW), although there are contradictory reports as well (5).

DHEAS, the major biochemical hallmark of adrenarche, has been considered as an antiatherogenic steroid, though unproven (6, 7, 8, 9, 10). The impact of DHEAS on atherogenesis was shown to have gender-specific influence in *in vitro* studies with human monocyte-derived macrophages (MDMs) due to differences in androgen receptor densities between sexes (8). Studies in rodents favor DHEAS as an antiatherogenic androgen (9, 10). In human male MDMs, DHEA-induced lipid accumulations resulting in foam cell formation, which is a critical step in atherosclerosis, were shown. However, this was not shown in females owing to scant androgen receptors on human female MDMs (11). Both DHEA and DHEAS serve as sex hormone precursors that are converted to more potent androgens and/or estrogens in peripheral tissues. Estrogens inhibit monocyte endothelial adhesion and inhibit endothelial cell expression of several adhesion molecules (12), which are necessary initial steps in atherosclerosis.

We hypothesized that PA in girls born appropriate for gestational age (AGA) may not be a risk factor for atherosclerosis when intrauterine growth restriction, changes in body mass index (BMI), and waist circumference (WC) as reliable indices of adiposity are excluded, as these are well-established risk factors for atherosclerosis in non-PA children as well. In order to test our hypothesis, we carried out a cross-sectional study by evaluating girls with PA born AGA and BMI- and WC-matched prepubertal peers born AGA.

## Materials and methods

### Subjects

The study group comprised 114 girls. For the girls with PA, the inclusion criteria were presence of pubic/axillary hair before the age of 8 years. Excluded from the study were girls who were born small for gestational age (SGA), children with chronic disease, genetic syndromes, congenital adrenal hyperplasia, and other endocrine abnormalities. Between November 2010 and January 2012, all eligible girls attending our pediatric endocrine clinic were invited to participate in the study. Among 94 girls with premature onset of pubic/axillary hair, after exclusion of steroidogenic enzyme defects and virilizing tumors by biochemical analyses (standard ACTH test) and adrenal ultrasonography, 69 (73.4%) were found to be eligible for inclusion to our study. The rest of the girls were either born SGA (*n*=22) or were diagnosed with nonclassical congenital adrenal hyperplasia (*n*=1), central puberty (*n*=1), or adrenocortical carcinoma (*n*=1). Forty-five BMI- and WC-matched peers born AGA were recruited consecutively from prepubertal girls followed in our Well Child Care Clinic. Data related to family history of CAD were retrieved from medical records. The controls enrolled in the study had no chronic disease and there was no history of CAD in three successive generations. Written consent was obtained from parents and assent was obtained from children over the age of 7 years. The study was approved by the Local Ethics Committee.

### Clinical evaluation

Weight and height of all the girls were measured by the same physician (A Uçar) using a calibrated Harpenden Stadiometer and an electronic scale (sensitivity at 0.1 kg level) respectively. BMI was calculated using the formula weight/(height)^2^. SDS of these measurements were calculated using national data (13, 14, 15). WC was measured to the nearest centimeter with a flexible steel tape measure while the subjects were in the standing position at the end of gentle expiration. The following anatomical landmarks were used: laterally, midway between the lowest portion of the rib cage and the iliac crest, and anteriorly midway between the xiphoid process of the sternum and the umbilicus (16). Obesity was defined as BMI SDS over 2. The WC values ≥90th percentile for age and gender were used to identify children and adolescents with abdominal obesity in accordance with the International Obesity Task Force cutoff values for obesity (17). Pubertal assessment was done by two pediatric endocrinologists (F Darendeliler and A Uçar) and development staged according to Tanner staging (18). BW data were retrieved from medical records and they were converted to SDS using national data (19). Systolic blood pressure (SBP) and diastolic blood pressure (DBP) measurements were based on the average of three repeated readings using a standard sphygmomanometer and their SDS values were calculated (20). Skeletal maturation was assessed by senior pediatric endocrinologists (F Darendeliler and N Saka) on the basis of roentgenograms of the left hand and wrist, evaluated according to Greulich & Pyle (21), with BA SDS calculated accordingly.

### Laboratory evaluation and biochemical assays

Fasting blood samples for glucose and lipids (total cholesterol, high-density lipoprotein cholesterol (HDL-C), low-density lipoprotein cholesterol, and triglycerides (TGs)), serum insulin, LH, FSH, DHEAS, total testosterone, and high-sensitivity C-reactive protein (hs-CRP) were drawn between 0830 and 1000 h, after an overnight fast. An oral glucose tolerance test (OGTT) was performed by administering 1.75 g/kg glucose (maximum 75 g) to each subject with samples for glucose and insulin analyses taken at 30, 60, 90, and 120 min. Determination of glucose and lipids was conducted on the same day. After separation, serum samples were frozen immediately and stored at −80 °C until assayed. Serum glucose levels over 99 mg/dl were accepted as impaired fasting glucose (IFG) (22). To determine insulin sensitivity (IS), homeostasis model assessment of insulin resistance (HOMA-IR) (fasting glucose (mg/dl)×insulin (mU/l)/405) (23) and IS index (ISI_comp_) (Matsuda index=10 000/square root of ((fasting glucose×fasting insulin)×(mean glucose×mean insulin during OGTT))) (24) were calculated. For calculation of mean serum glucose and mean serum insulin (MSI) during the OGTT, the area under the glucose and insulin curves was assessed according to the trapezoidal rule. HOMA-IR >2.5 was used to define IR (25).

Evaluation of lipids included estimation of atherogenic index (AI; TG/HDL-C) (26). Because universally accepted criteria to estimate metabolic risks in children are lacking, we included the same risk factor variables that are used for the adult definition. Dyslipidemia was defined as serum HDL-C concentrations ≤40 mg/dl and serum TG concentrations ≥110 mg/dl (27).

The assays were routinely performed by a semiautomated Immulite System and commercial kits obtained from Diagnostic Products Corporation (DPC; Los Angeles, CA, USA), were used. Serum glucose was measured using an automated glucose oxidase method. Serum LH and FSH concentrations were measured by immunochemiluminometric assay. DHEAS and testosterone levels were measured by RIA and insulin levels by ECL assay. hs-CRP levels were measured using a hs-CRP assay (Behring Latex-Enhanced using the Behring Nephelometer BN-100; Behring Diagnostics, Westwood, MA, USA). The sensitivity of the assay ranged from 0.04 to 5.0 mg/l. LH and FSH assays had intra-assay coefficient of variations (CV) 4.8 and 7.5% and interassay CV of 10.7 and 5.4% respectively. Sensitivity of the testosterone assay was 0.05 ng/ml, the intra-assay CV was 4.5%, and the interassay CV was 6.4%. Values for the DHEAS assay were 4.5% (intra-assay CV) and 5.5% (interassay CV). Sensitivity of the insulin assay was 0.05 mU/l. Intra-assay and interassay CV were <5%. Plasma lipids were measured by Integra-800 autoanalyzer (Roche).

### Comparison between groups

Girls with PA and controls were compared with respect to anthropometric and laboratory data. The presence of significant correlations between anthropometric data, plasma lipids, hs-CRP, HOMA-IR, IS index, and hormonal measurements within each study group was assessed.

### Statistical analysis

Normality of the variables was tested by D'Agostino and Pearson's Omnibus normality test. The results are presented as mean±s.d. or median (interquartile range). Student's *t*-test was used for comparison of variables between groups, and nonparametric tests were used if skewed variables do not have Gaussian distribution after transformation. Comparison of categorical variables was performed using *χ* ^2^ test and Fisher's exact test. Relative risk (RR) was used to calculate the risk coefficient of significant quantitative data with 95% confidence intervals (95% CIs) given at a statistical significance of *P*<0.05. Correlations between continuous variables were evaluated using Pearson's correlation analyses. In controls, the nonlinear correlations between age and serum DHEAS and testosterone concentrations were calculated and graphically demonstrated by nonlinear regression analysis using GraphPad Prism Software (San Diego, CA, USA). Multivariate regression analysis tested the independent association and contribution of changes in BMI, WC, IS index, serum DHEAS, hs-CRP, and OGTT-derived parameters with the dependent variable (changes in AI). Before regression analysis, variables that did not follow a normal distribution (DHEAS, HOMA-IR, and WC) were log transformed. The results were evaluated at the 95% CI and significance was granted for *P*<0.05. All analyses were performed using SPSS 15 (SPSS, Inc., Chicago, IL, USA) and GraphPad Prism 5 Software.

## Results

The mean age of the girls with PA and controls was similar as planned in the study design (7.1±1 vs 7.5±1.9 years respectively, *P*>0.05). Six of the girls with PA were older than 8 years at evaluation (range 8.3–9.2 years). The median age of the girls with PA at onset of pubarche and at evaluation was 6.6 years (25–75%; interquartile range (IQR) 6–7.2 years) and 7.1 years (25–75%; IQR 6.3–7.7 years) respectively. BW SDS were similar in both groups as we expected (*P*>0.05; Table 1). Family history of CAD was present in 22 (31.8%) girls with PA. None of the girls in controls had a family history of CAD.

### Clinical data

SDS values for weight and height in girls with PA were significantly higher than those of the controls (*P*=0.008, *P*=0.0006 respectively), but BMI SDS and WC were similar between the groups (*P*>0.05), as planned in the study design (Table 1). In girls with PA and controls, BMI SDS values were consistent with obesity in 10.1 and 11.1% of the cases respectively (*P*=1), and abdominal obesity according to WC values was present in 11.6 and 15.5% of the subjects respectively (*P*=0.58). BA SDS was significantly higher in girls with PA than in controls (*P*=0.0002; Table 1). SBP SDS and DBP SDS were not significantly different in girls with PA and controls (*P*>0.05).

### Hormonal data

Serum LH and FSH concentrations were within prepubertal ranges in all the girls enrolled in the study and they were similar between girls with PA and controls (*P*>0.05; Table 2). Serum DHEAS and testosterone concentrations were significantly higher in girls with PA than the controls (*P*=0.0001 and *P*=0.031 respectively; Table 2). DHEAS concentrations were >40 μg/dl in all the girls with PA.

### Insulin sensitivity

The comparison of OGTT findings in girls with PA and controls is depicted in Table 2. IFG was present in 9 (13%; range 102–104 mg/dl) girls with PA and 7 (15.5%; range 100–106 mg/dl) girls in controls. The risk coefficient for having IFG was not statistically significant (*P*=0.78). HOMA-IR was significantly higher in girls with PA than in controls (*P*<0.05). However, at a cutoff level of 2.5, the groups were not significantly different with respect to the presence of IR (*P*>0.05). ISI_comp_ was significantly lower in girls with PA than in controls (*P*=0.0009).

### hs-CRP and fasting plasma lipids

hs-CRP concentrations were similar in girls with PA and controls (*P*>0.05; Table 2). HDL-C concentrations were significantly higher in girls with PA than in controls (*P*<0.0001; Table 2). HDL-C concentrations were ≤40 mg/dl in 7 (10.1%) girls with PA and 16 (23.1%) controls. The risk for having HDL ≤40 mg/dl was higher in controls than in girls with PA (RR=2.2 (95% CI), 1.2–4.2; *P*=0.0016). Serum TG concentrations ≥110 mg/dl were found in 4 (5.7%) girls with PA and 9 (13%) controls. The risk for having serum TGs ≥110 mg/dl was higher in controls than in the girls with PA (RR=2.1 (95% CI), 0.9–4.8; *P*=0.032). AI was significantly lower in girls with PA than in controls (*P*<0.001). When we compared AI levels in girls with PA having history of CAD in family with those who had no such history, the differences in AI levels between the subgroups were not significant (1.45±0.72 vs 1.4±0.53, *P*=0.758 respectively).

### Correlations between parameters

#### Girls with PA

Weight SDS, BMI SDS, and WC had significant correlations with fasting serum insulin (*r*=0.600, *P*=0.0001; *r*=0.470, *P*=0.004; *r*=0.441, *P*=0.001 respectively), HOMA-IR (*r*=0.560, *P*=0.001; *r*=0.583, *P*=0.001; *P*=0.597, *P*<0.0001 respectively), ISI_comp_ (*r*=−0.700, *P*=0.0001; *r*=−0.687, *P*=0.0001; *r*=−0.596, *P*=0.0001 respectively), serum TGs (*r*=0.361, *P*=0.01; *r*=0.423, *P*=0.004; *r*=0.392, *P*=0.009 respectively), HDL-C (*r*=−0.300, *P*=0.05; *r*=−0.472, *P*=0.002, *r*=−0.41, *P*=0.02 respectively), and hs-CRP (*r*=0.749, *P*<0.0001, *r*=0.733, *P*<0.0001, *r*=0.730, *P*<0.0001 respectively). Age at evaluation and the anthropometric measurements did not significantly correlate with serum DHEAS and testosterone concentrations (data not shown). Correlations of AI with clinical data and laboratory-related parameters are depicted in Table 3. The correlation of OGTT-derived parameters with AI remained significant after adjustment for BMI (data not shown). The significant correlation of AI with ISI_comp_ (*r*=−0.38, *P*=0.001) disappeared after correcting for BMI SDS and weight SDS (*r*=−0.185, *P*=0.16). Multivariate regression analysis including variables depicted in Table 4 revealed that increase in serum DHEAS concentrations were significantly and independently associated with decrease in AI in girls with PA (*R* ^2^ =0.475, *β*=−0.018, *P*=0.0001).

#### Controls

**Figure 1:**
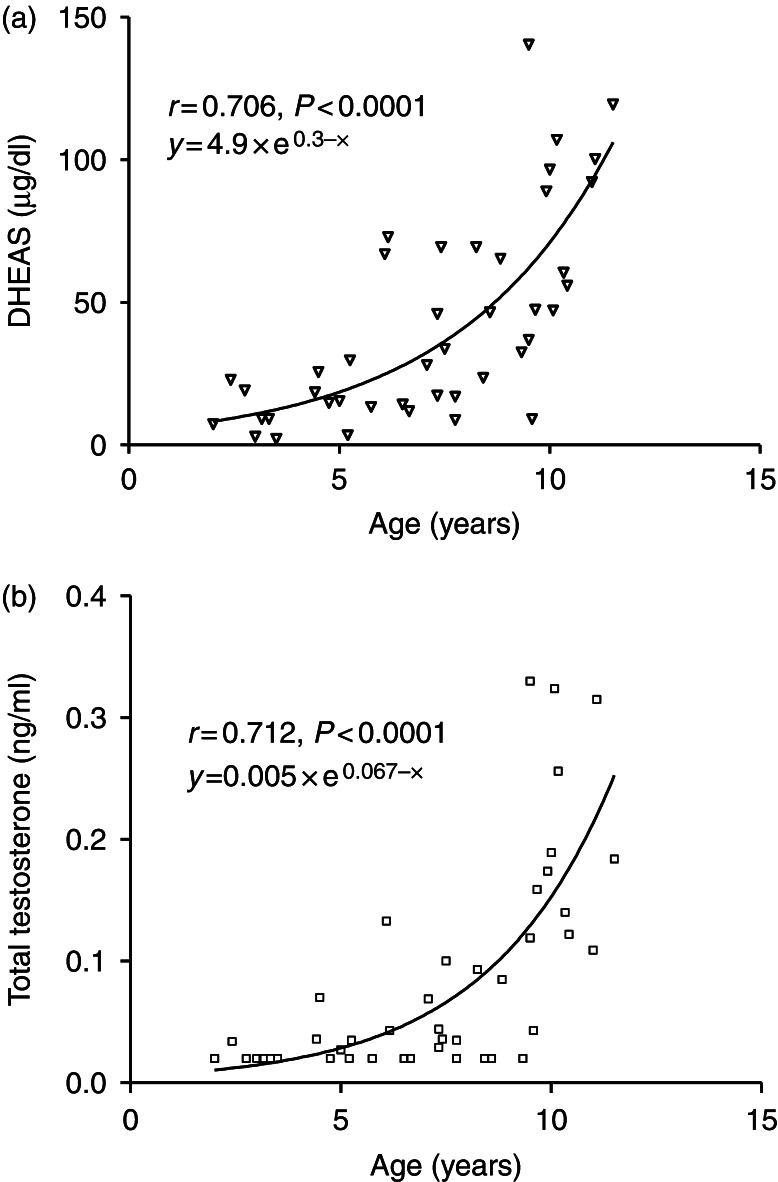
The correlations of serum DHEAS (a) and total testosterone (b) concentrations with age in controls (n=45). Note the exponential increase in DHEAS and testosterone concentrations with age.

Correlation analyses of the anthropometric measurements with laboratory-related parameters yielded similar findings in controls as in girls with PA (data not shown). Serum DHEAS and testosterone concentrations significantly correlated with age (*r*=0.706, *P*<0.0001; *r*=0.712, *P*<0.0001, respectively; Fig. 1).

## Discussion

Owing to the multifaceted nature of atherosclerosis, we selected BW-, BMI-, and WC-matched controls to adjust for factors that also contribute to atherosclerosis in non-PA girls to better elucidate the independent impact of PA on the issue. The distribution of obese girls in controls was similar to those of recent publications from Turkey in accordance with the increasing prevalence of obesity worldwide (28, 29).

Studies insofar have focused on the possible link between reduced fetal growth, dyslipidemia, IR, and PA ((30, 31, 32, 33), also reviewed in (5)). Although a large body of evidence – especially from studies in Catalonian girls with PA – suggests a possible link between these issues, there are also some contradictory reports with respect to dyslipidemia (1, 34, 35, 36), IR (34), reduced BW (1, 36, 37), and atherosclerosis (38) in girls with PA. One of the reasons for the discrepancy of the findings in studies published so far is that subjects were selected as controls if they had either normal fasting plasma lipids (38, 39) or normal/low normal BMI (1, 34, 40) or they were selected with no information given in relation to BW (34, 38, 39, 41). Moreover, in some of these studies, the subjects had PA by history (41), or the studies included relatively smaller number of subjects (34, 38, 39, 40, 41) or no information was given in relation to whether precocious pubarche was accompanied by PA in all the subjects included in the study (35, 39, 40). Our findings in addition to those published so far also support the statement that PA is a heterogeneous condition with clinical characteristics being dependent on ethnicity (42) and some yet unknown factors.

A novel finding of our study is that prepubertal girls with PA born AGA have reduced AI when compared with BMI-, WC-, and BW-matched controls. Girls with PA had significantly higher serum HDL-C concentrations and lower serum TG concentrations when compared with controls (*P*<0.05). Indeed, HDL-C concentrations in children have been inversely correlated with the incidence of CAD in the different countries studied (43). When we evaluated clinical and laboratory-related variables shown to have correlations with AI, we found that DHEAS was the major factor associated with a reduction in AI (TG/HDL-C; *R* ^2^=0.475, *β*=−0.018, *P*<0.0001). The contribution of reduced IS to increased AI disappeared after correction for changes in BMI and WC, indicating the utmost importance of change in body fat in atherosclerosis. The mechanism by which DHEAS may account for decrease in AI is not clear in our study. *In vitro* experiments may not represent the same conditions as in the arterial wall *in vivo*. In the *in vivo* context, the ‘net’ vascular biological effect of DHEA may be the summation of both direct and indirect effect via androgenic and estrogenic metabolites. We speculate that although adrenal androgen concentrations in girls with PA are higher than their BMI- and WC-matched peers, they cannot exert their proatherogenic effect in girls due to sparsity of androgen receptors on female human MDMs and also due to aromatization of the slightly increased androgens to estrogens and their metabolites in the periphery takes place. We also speculate that this seemingly unfavorable androgen profile in girls with PA when compared to BMI- and WC-matched peers is probably overcome by the presence of androgen-insensitive female MDMs and conversion of these ‘macrophage inert’ androgens to estrogens in the periphery, giving rise to the endocrine paradox.

The novelty of the methodology chosen in recruitment of subjects in our study is that we excluded girls with PA born SGA as it is postulated that a transient prenatal growth constraint in children born SGA may be followed by a permanent resetting of endocrine axes. Meta-analysis of all published studies revealed that low BW is indeed associated with type 2 diabetes, which is linked to atherosclerosis and metabolic syndrome (44). Nutritional factors do not seem to play a role in early onset of adrenarche in our cohort as DHEAS and testosterone concentrations failed to correlate significantly with BMI and WC. In controls, we found that DHEAS and testosterone significantly correlated with age unlike the case in girls with PA, where this correlation was not significant. These findings support an intra-adrenal etiology in earlier onset of adrenarche in girls with PA born AGA.

Although we found that girls with PA had lower IS and higher MSI concentrations during OTT, they were not found to be more IR than controls unlike the findings in Catalonian girls, in which the presence of dyslipidemia, IR, and atherogenesis was linked to reduced fetal growth (30, 31, 32, 33). The presence of IR in girls with PA may require reassessment as a recent consensus on evaluation of IR in children declared that none of the currently used parameters be considered as reliable and that new studies be carried out to define IR in children (45).

We measured WC of all the girls to evaluate abdominal obesity as it is a strong correlation of total body fat (46), which is clearly associated with development of atherosclerosis and metabolic syndrome. Recently, Catalonian girls with PA were shown to have increased WC measurements compared with matched controls, and the increase in abdominal fat was significantly correlated with reduction in BW (47). In our study, we eliminated a pivotal contributor to atherosclerosis by selecting AGA born girls, and both BMI and WC measurements were similar between girls with PA and controls. History of being born SGA was associated with increased abdominal fat (48). Therefore, these selection criteria along with ethnicity may account for the discrepancy between our study and that of Ibanez *et al*. (47).

hs-CRP has been shown to be a good marker of cardiovascular disease in obese children and adolescents (49). In girls with PA, we found that hs-CRP significantly correlated with BMI SDS and WC, and there was no significant difference in hs-CRP concentrations between girls with PA and controls. This finding suggests that subclinical inflammation is not increased in girls with PA born AGA at initial evaluation when compared with controls, and the increase in hs-CRP is associated with increase in BMI and WC as in non-PA girls. This finding is important as inflammation is one of the well-established factors related to vascular endothelial dysfunction. A recent study comparing carotid ultrasound findings of girls with PA and BMI-matched controls found no significant difference with respect to carotid intima-media thickness and in systolic inner and outer diameters despite the fact that controls were chosen among BMI-matched subjects who had normal fasting plasma lipids (38).

In addition to the strengths of our study outlined earlier, three limitations of our study merit comment. First, all the girls in our study were prepubertal, and it can be speculated that metabolic changes may be observed later in the process and not necessarily at the time of diagnosis as our study is cross-sectional. However, clinical studies in children have demonstrated that an atherogenic pattern of risk factors can start in childhood (50, 51), and both the girls with PA and controls included obese subjects to make adjustment for the impact of adiposity on atherogenesis. We also did not assess body composition of the subjects in our cohort, but WC was proven to be a reliable measure of adiposity in children (46). Thirdly, the absence of a reliable screening tool to assess IR in children makes it difficult to interpret results in relation to indices of IR.

Our preliminary results suggest that metabolic problems in girls with PA born AGA be related to changes in adiposity. PA may be an independent negative risk factor for atherogenesis at initial assessment when compared with non-PA subjects having similar adiposity. Reduced atherogenic indices in girls with PA born AGA may be related to increased secretion of DHEAS from the adrenals. *In vivo* studies involving end-products of peripheral metabolism of DHEAS will probably highlight this endocrine paradox. Whether the favorable impact of PA in AGA born girls on atherogenesis persists in puberty and adulthood remains to be elucidated.

## Figures and Tables

**Table 1:** Birth weight and clinical data of the girls with precocious adrenarche (*n*=69) and controls (*n*=45).

	**Girls with PA** (*n*=69)	**Controls** (*n*=45)
	Mean±s.d. (median)	Mean±s.d. (median)	***P***
Birth weight SDS	−0.4±1 (−0.6)	−0.3±0.9 (−0.7)	0.42
Anthropometric data (SDS)			
Weight	0.6±1 (0.6)	0±0.2 (−0.2)	0.008*
Height	0.4±1 (0.4)	−0.3±1.1 (−0.4)	0.0006*
BMI	0.5±1.2 (0.5)	0.2±1.1 (−0.1)	0.13
Waist circumference (cm)	55.7±3.7 (55)	54.7±4.8 (53.6)	0.22
Bone age SDS	0.6±1 (1)	0±1 (0)	0.0002*
Systolic blood pressure SDS	0±0.7 (0)	0±0.9	0.61
Diastolic blood pressure SDS	0.2±0.6 (0)	0±0.7 (0)	0.30

*Statistically significant.

**Table 2:** Comparison of the laboratory-related parameters in girls with precocious adrenarche (*n*=69) and controls (*n*=45).

	**Girls with precocious adrenarche** (*n*=69)	**Controls** (*n*=45)
	Mean±s.d. (median)	Mean±s.d. (median)	***P***
hs-CRP (mg/l)	0.85±1 (0.4)	0.94±1.1 (0.6)	0.56
Hormonal data			
LH (IU/l)	0.13±0.05 (0.1)	0.12±0.04 (0.11)	0.83
FSH (IU/l)	1.53±0.77 (1.3)	1.81±1.04 (1.8)	0.28
DHEAS (μg/dl)	68.3±18.3 (64)	44.6±41.4 (29.7)	***0.0001***
Total testosterone (ng/ml)	0.18±0.19 (0.1)	0.08±0.09 (0.04)	***0.031***
Insulin sensitivity			
fP-glucose (mg/dl)	81.7±9.5 (79)	83.3±8.7 (80)	0.69
fS-insulin (mU/l)	7.8±3.5 (6.2)	6±3.6 (5.6)	***0.0065***
HOMA-IR	1.6±0.9 (1.3)	1.3±0.9 (1)	***0.0009***
2-h glucose (mg/dl)	106.4±8.2 (107)	103.2±10.7 (105)	0.11
Mean-S-insulin (mU/l)	38±9.1 (33)	31.5±6.5 (29)	***0.023***
ISI_comp_	0.83±0.12 (0.84)	0.92±0.16 (0.96)	***0.0009***
Fasting plasma lipids			
TC (mg/dl)	166±35	164±22	0.69
HDL-C (mg/dl)	54.1±10.3 (54.5)	45±13.6 (44)	***0.0001***
LDL-C (mg/dl)	87±20 (83)	103±31 (103)	***0.0004***
TG (mg/dl)	74.4±33.4 (69)	91.9±46.6 (87)	***0.0208***
Atherogenic index (TG/HDL-C)	1.44±0.67 (1.33)	2.08±1.14 (1.74)	***0.0003***

hs-CRP, high-sensitivity C-reactive protein; f, fasting; p, plasma; HOMA-IR, homeostasis model assessment of insulin resistance; mean-S-insulin, mean serum insulin measured during the OGTT; ISI_comp_, insulin sensitivity index; TC, total cholesterol; HDL-C, high-density lipoprotein cholesterol; LDL-C, low-density lipoprotein cholesterol; TG, triglyceride; HOMA-IR=(fasting insulin (μU/ml)×fasting glucose (mg/dl))/405; ISI_comp_=10 000 square root of (fasting glucose (mg/l)×fasting insulin (μU/l)×mean glucose (mg/dl)×mean insulin (μU/l)). Bold and italic *P* values indicate significant differences (*P*<0.05) in the results between the two study groups.

**Table 3:** Correlations of atherogenic index with clinical and laboratory-related parameters in girls with precocious adrenarche (*n*=69).

	**Atherogenic index** (triglycerides/HDL-C)	
	*r*	*P*
Clinical data		
Birth weight SDS	−0.05	0.69
Chronological age (years)	0.03	0.82
Weight SDS	***0.41***	***0.001***
BMI SDS	***0.44***	***0.001***
Waist circumference (cm)	***0.39***	***0.001***
Bone age SDS	***0.38***	***0.001***
SBP SDS	0.08	0.54
DBP SDS	0.04	0.76
Laboratory-related parameters		
hs-CRP	**0.29**	***0.015***
DHEAS (μg/dl)	***−0.58***	***0.001***
Total testosterone (ng/ml)	−0.06	0.826
HOMA-IR	***0.27***	***0.023***
2-h glucose (mg/dl)	***0.27***	***0.023***
Mean-S-insulin (mU/l)	0.21	0.093
ISI_comp_	***−0.38***	***0.001***

HDL-C, high-density lipoprotein cholesterol; *r*, Pearson's correlation coefficient; BMI, body mass index; SBP, systolic blood pressure; DBP, diastolic blood pressure; HOMA-IR, homeostasis model assessment of insulin resistance; mean-S-insulin, mean serum insulin concentration during the OGTT; ISI_comp_, insulin sensitivity index. Bold and italic data indicate statistically significant correlations (*P*<0.05).

**Table 4:** Multiple regression analysis of the factors that influenced the atherogenic index in girls with precocious adrenarche born appropriate for gestational age (*R* ^2^=0.475).

	***B***	**s.e.m.**	***β***	***t***	***P***
Constant	3.327	1.953		1.703	0.094
Weight SDS	−0.135	0.177	−0.235	−0.764	0.448
Body mass index SDS	0.226	0.172	0.446	1.313	0.194
Waist circumference	−0.018	0.029	−0.117	−0.632	0.530
hs-CRP	−0.112	0.137	−0.198	−0.546	0.268
DHEAS	−0.018	0.003	−0.556	−5.587	***0.0001***
HOMA-IR	−0.078	0.086	−0.114	−0.905	0.369
ISI_comp_	−0.587	0.700	−0.120	−0.839	0.405
Mean-S-insulin	−0.019	0.011	−0.273	−1.807	0.086
2-h glucose	0.008	0.009	0.107	0.901	0.371

hs-CRP, high-sensitivity CRP; HOMA-IR, homeostasis model assessment of insulin resistance; ISI_comp_, insulin sensitivity index; mean-S-insulin, mean serum insulin concentration measured during the OGTT.
